# Efficacy of electric-powered cleaning instruments in edentulous patients with implant-supported full-arch fixed prostheses: a crossover design

**DOI:** 10.1186/s40729-019-0164-8

**Published:** 2019-03-26

**Authors:** Toru Maeda, Taro Mukaibo, Chihiro Masaki, Sirapat Thongpoung, Shintaro Tsuka, Akiko Tamura, Fumiko Aonuma, Yusuke Kondo, Ryuji Hosokawa

**Affiliations:** Division of Oral Reconstruction and Rehabilitation, Kyushu Dental University, 2-6-1 Manazuru, Kokurakita-Ku, Kitakyushu City, Fukuoka 803-8580 Japan

**Keywords:** Oral hygiene, Manual toothbrush, Electric toothbrush, Dental implant, Implant-supported full-arch fixed prosthesis, All-on-4

## Abstract

**Background:**

The aim of this study was to evaluate the plaque removal efficacies of electric toothbrushes and electric dental floss compared with conventional manual toothbrushing in cleaning the fitting surface of an All-on-4™ concept (Nobel Biocare, Zürich-Flughafen, Switzerland) implant-supported fixed dental prosthesis (FDP).

**Methods:**

Nine patients with maxillary edentulous arches participated in the study. We investigated two electric-powered brushes (Sonicare Diamond Clean®, Koninklijke Philips N.V., Amsterdam, the Netherlands [SD group], and the Oral-B Professional Care Smart Series 5000®, Braun GmbH, Kronberg, Germany [OralB group]) and one electric dental floss unit (Air Floss®, Koninklijke Philips N.V. [AF group]). A manual toothbrush (Tuft24® MS, OralCare Inc., Tokyo, Japan) was used by the control group. The fitting surface of the FDP was stained to allow visualization of the entire accumulated plaque area. Both the buccal and palatal portions of the plaque area were assessed before and after brushing to evaluate each instrument’s plaque removal rate using a crossover study design. Two-week washout periods were employed between each evaluation.

**Results:**

The plaque removal rates were 53.5 ± 8.5%, 70.9 ± 6.5%, 75.4 ± 6.3%, and 74.4 ± 4.2% for the control, AF, OralB, and SD groups, respectively. When participants were divided into two groups based on their plaque removal rates with a manual toothbrush (poor brushing and good brushing), the poor brushing group showed significant improvement in the plaque removal rate when using electric-powered toothbrushes. The plaque removal rates for the buccal area were significantly higher for the OralB and SD groups than for the manual brushing group (control group), with rates of 52.8 ± 7.9%, 70.1 ± 7.3%, 77.7 ± 6.5%, and 79.5 ± 3.7% for the control, AF, OralB, and SD groups, respectively. The plaque removal rates in the palatal area were consistently lower than those in the buccal area for each of the three electric instruments.

**Conclusions:**

The results suggest that patients who are not adept at manual toothbrushing may potentially improve their removal of plaque from the fitting surfaces of FDPs by using electric toothbrushes.

## Background

Oral hygiene is important for the long-term stability of dental implants and prevention of biological complications associated with implants. Implant-supported fixed dental prostheses (FDPs) based on the All-on-4™ treatment concept (Nobel Biocare, Zürich-Flughafen, Switzerland) have been developed [1], and their success and survival rates have been widely reported [2–4]. It has also been reported that fixed prostheses can contribute to the improvement of oral health-related quality of life in patients wearing complete dentures [5]. However, it is often difficult for patients who wear fixed prostheses to remove plaque from the fitting surface of the prostheses because they are frequently placed in close contact with the alveolar ridge in the maxilla to reduce speech or esthetic issues. There have been studies on the association between plaque accumulation and peri-implant mucositis [6, 7]. Insufficient oral hygiene is likely associated with peri-implantitis [8], and inadequate plaque control and inflammation can lead to compromise of the osseointegration of implants [9] or minor complications such as soft tissue recession [10] or halitosis [11]. Although plaque accumulation on the fitting surface of the All-on-4™ concept FDPs does not immediately contribute to inflammation around the implants, and thus may not be directly associated with peri-implantitis, we believe that effective plaque removal on the fitting surface is indispensable to the maintenance of oral hygiene, which could be associated with the inflammation of peri-implant tissues.

To date, there have been a limited number of studies on maintenance protocols for implant-supported FDPs [12, 13], and there is inadequate evidence available regarding self-performed oral hygiene in patients with FDPs [14, 15]. Thus, the aim of this study was to evaluate the plaque removal efficacies of electric toothbrushes and electric dental floss compared with conventional manual toothbrushing in cleaning the fitting surface of an All-on-4™ concept FDP.

## Methods

Nine maxillary edentulous patients (seven males, two females) with a mean age of 68.0 ± 2.7 years (range 55–81 years) were recruited and voluntarily participated in this crossover study, which was conducted at Kyushu Dental University Hospital’s implant dentistry department. The implant surgery and fabrication of the prostheses were performed in accordance with the All-on-4™ treatment concept [1], in which a screw-retained provisional prosthesis is seated on four straight or angulated (17° and 30°) multiunit abutments (Nobel Biocare, Zürich-Flughafen, Switzerland) that are connected to the implants immediately after surgery. Computer-aided design/computer-aided manufacturing was used to fabricate the prostheses using a titanium-milled frame bonded with composite resin. The fitting surface of the prosthesis was fabricated in an ovate shape with contacts intimate to the residual alveolar ridge. After placement of the final prostheses, patients were verbally instructed to use their own manual toothbrushes to clean the boundary between the prosthesis and the alveolar ridge three times per day. As a routine follow-up cleaning protocol, each patient was recalled every 3 months, and the prosthesis was removed and cleaned with a toothbrush (Tuft24®MS, OralCare Inc., Tokyo, Japan) under running water and sterilized in an ultrasonic bath filled with chlorhexidine 0.05% (0.05 *w*/*v* MASKIN®/water, Maruishi Pharmaceutical Co., Ltd., Osaka, Japan) for 5 min. The study started 3 months after the final follow-up, at which point each participant had worn the final prosthesis for at least 1 year.

**Fig. 1:**
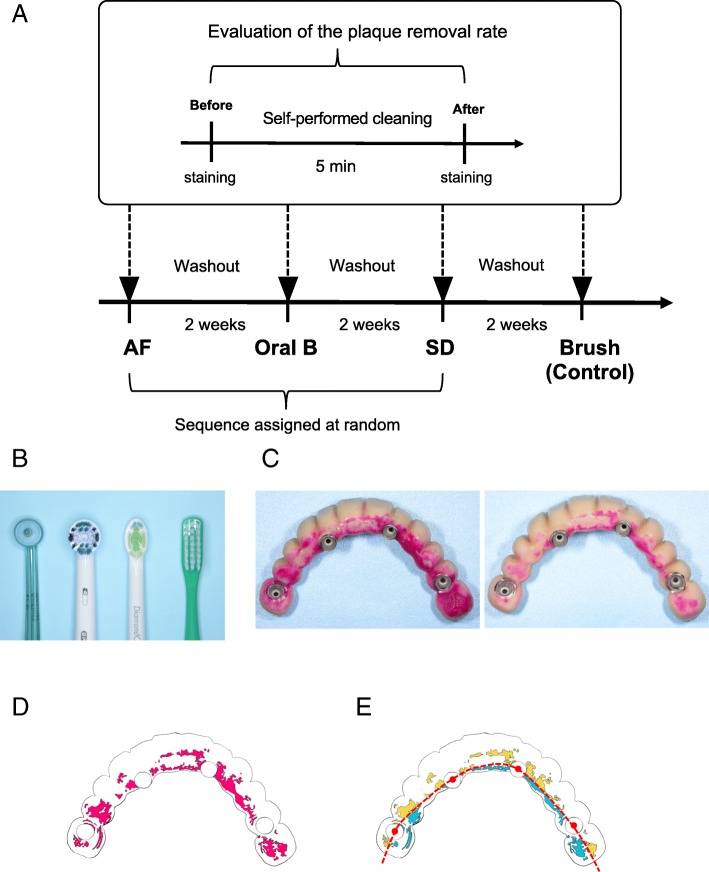
Study design and digital images for the comparison of four different tooth-cleaning instruments. **a** Each patient was randomly assigned to use two electric-powered brushes (OralB and SD) and one electric dental floss unit (AF). A manual toothbrush was used for the control group. **b** Brush heads of each instrument. From left to right: AF (nozzle HX8002/05), OralB (brushing head EB20), SD (brushing head HX6074/05), and Brush (Tuft24® MS). **c** Left: fitting surface of an implant-supported FDP before brushing; right: after brushing. Plaque is stained red. **d** The plaque area and the outline of the FDP in **c** were traced manually, and the plaque was colored with pink to calculate the area before and after brushing (only after brushing is shown in the figure). **e** The plaque area in **d** was divided into buccal and palatal sides with a dotted line that passes through the center points of each of the four implants’ midpoints. The yellow area denotes buccal side plaque, and the light blue area denotes palatal side plaque

We investigated two electric-powered brushes (Sonicare Diamond Clean® attached to an HX6074/05 brushing head, Koninklijke Philips N.V., Amsterdam, the Netherlands [SD group], and the Oral-B Professional Care Smart Series 5000® attached to an EB20 brushing head, Braun GmbH, Kronberg, Germany [OralB group]) and one electric dental floss unit (Air Floss® attached to an HX8002/05 nozzle, Koninklijke Philips N.V. [AF group]). A manual toothbrush (Tuft24® MS, OralCare Inc., Tokyo, Japan) was used for the control group. Dentifrice was not used during the assessments in order to solely evaluate the ability of the instruments. To evaluate the efficacy of each brushing instrument, three electric instruments were randomly assigned for use along with 2-week washout periods between evaluations; the manual toothbrush was used last and served as the control. In this crossover study design, each instrument was used one time for 5 min during the respective assessment, and each participant used all four cleaning instruments (Fig. 1a, b). All participants confirmed that they had not been using any electric tooth cleaning instruments; thus, they used their own manual toothbrushes during the washout periods. The randomization sequence for the three electric instruments was created using Excel 2011 (Microsoft, Redmond, WA, USA). The study procedure was approved by the Kyushu Dental University Ethics Committee (approval number 13-3) and followed the guidelines of the amended Declaration of Helsinki. All participants provided written informed consent.

First, the fixed maxillary prosthesis was removed, and the plaque that had accumulated on the fitting surface was dyed with plaque-disclosing solution (Dent. liquid plaque tester, Lion Corp., Tokyo, Japan). Photographs of the dyed fitting surface were taken with a digital camera (D5200, Nikon Corp., Tokyo, Japan) (Fig. 1c). After the prosthesis was placed back in the mouth and retained by 15-Ncm screws, the patient was instructed to brush for 5 min in front of a mirror. Subsequent to removal of the prosthesis, a photograph of the fitting surface was taken to evaluate the remaining plaque. Finally, the remaining plaque was removed with a manual toothbrush (Tuft24® MS) under running water, and the prosthesis was replaced using 15-Ncm screws. Patients were only given basic guidance on use of the electric instrument, such as how to turn it on and off.

To calculate the dyed plaque area, tracing paper was mounted on the photograph. The margins of the prostheses and the plaque areas were traced manually. Only one individual performed the tracing; this individual was blinded to assignment, in order to ensure consistent assessment. The encircled plaque area was filled with red color (Fig. 1d) using an open-source image editor (GUN Image Manipulation Program) [16], followed by identification and calculation of the colored area with an open-source image-processing program (ImageJ) [17]. To determine the percentage of the area covered with plaque within the fitting surface of the prosthesis, the sum of the plaque area was divided by the entire outlined area of the fitting surface. To compare the plaque removal rates among the four groups, plaque-covered areas were directly compared before and after cleaning. Participants were then divided into two groups based on the plaque removal rate using a manual toothbrush; those with plaque removal rates ≤ 60% were assigned to the poor brushing group, and those with rates > 60% were assigned to the good brushing group. The buccal and palatal areas of the prosthesis were defined by dividing the implant area at the center line that passed through each of the four implants’ midpoints (Fig. 1e).

Kruskal-Wallis one-way analysis of variance, followed by Dunn’s multiple comparisons test, was used to determine statistically significant differences. Unpaired *t* tests with Welch’s correction were performed to compare the two groups. Statistical software (Prism7 Software, GraphPad Software, La Jolla, CA, USA) was used for all analyses; *p* <  0.05 was considered statistically significant. All results are presented as mean ± standard error. Statistical power analysis was performed using an open-source power analysis program (G*Power 3.1) [18].

## Results

**Fig. 2:**
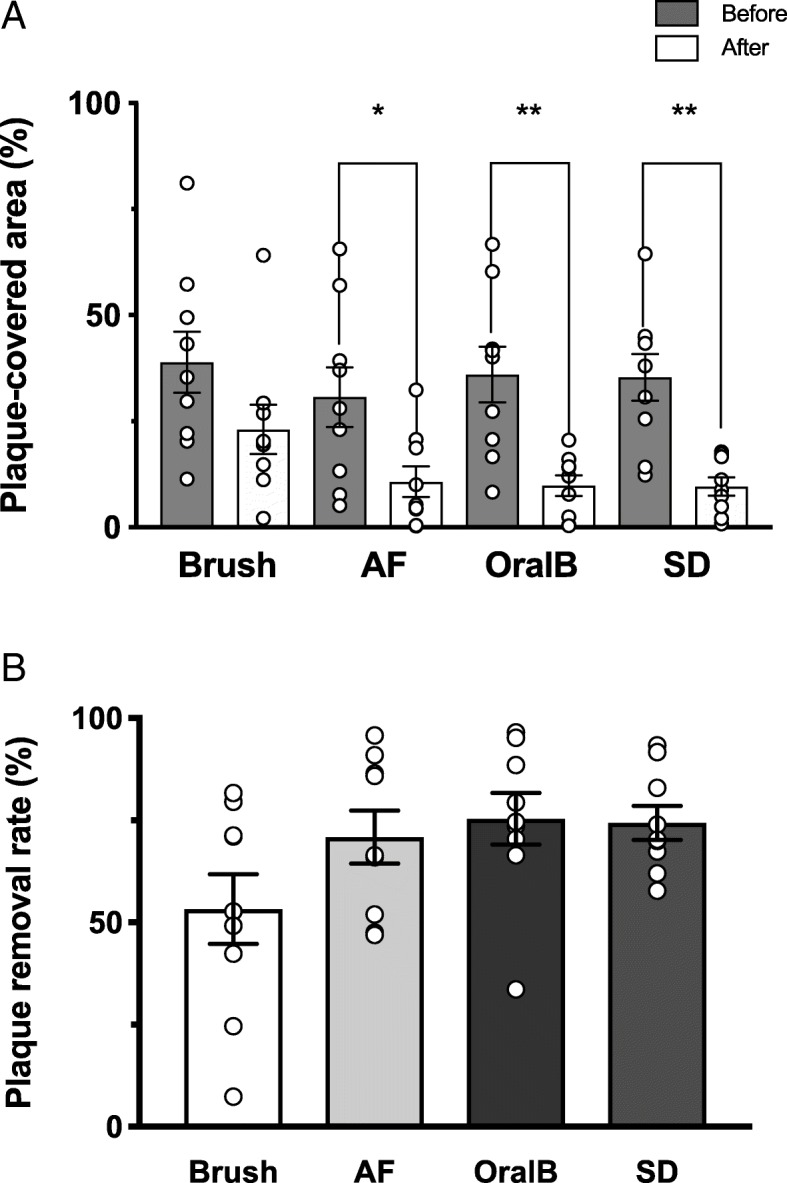
Plaque areas before and after brushing and plaque removal rates. **a** All brushing instruments except for the manual toothbrush showed significant reduction of accumulated plaque in the fitting surfaces of implant-supported fixed dental prostheses. Note that the plaque areas before brushing did not differ significantly among the four instruments based on multiple comparison statistics. **b** No significant differences in plaque reduction were observed among the four instruments. *p <  0 .05; **p <  0.005

**Fig. 3:**
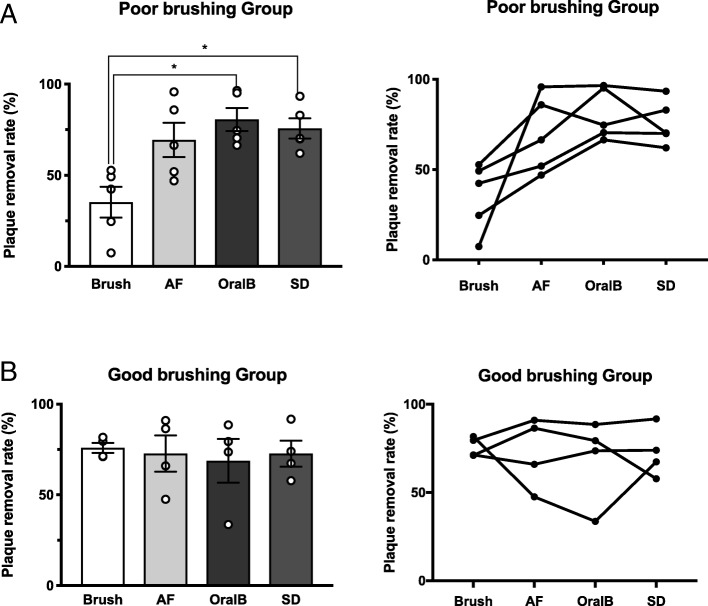
Comparison of plaque removal rates among groups according to manual toothbrushing skill. **a** Plaque removal rates of subjects with poor brushing skills (*n* = 5) improved significantly with use of electric toothbrushes, relative to the use of a manual toothbrush. **b** Consecutive data plots of the individual subjects represented in Fig. 2a. **c** Subjects with good toothbrushing skills (*n* = 4) did not exhibit significant differences among cleaning instruments. **d** Consecutive data of the individual subjects in the good brushing group. *p <  0.05

All brushing instruments except the manual toothbrush resulted in significant reduction of accumulated plaque after brushing (Fig. 2a). The total areas of average plaque accumulation at the fitting surface before and after cleaning were 35.2 ± 3.2% and 13.3 ± 2.1%, respectively (Table 1). The plaque removal rates were 53.3 ± 8.5%, 70.9 ± 6.5%, 75.4 ± 6.3%, and 74.4 ± 4.2% for the control, AF, OralB, and SD groups, respectively; no significant differences were observed among the four cleaning instruments (Fig. 2b). However, when participants were divided into poor and good brushing groups, the poor brushing group demonstrated significant improvement when using either the SD or OralB instruments (Fig. 3a); the good brushing group did not exhibit significant improvement with any of the four cleaning instruments (Fig. 3b).

**Table 1:** Plaque-covered areas before and after brushing and plaque removal rates

Instrument	Plaque-covered area			Plaque removal rate	
	Before (%)	After (%)	*p* value	Plaque removal rate (%)	Adjusted *p* vs. brush
All subjects (*n* = 9)					
Brush	38.9 ± 7.2	23.1 ± 5.8	0.107	53.3 ± 8.5	
AF	30.7 ± 7.1	10.7 ± 3.6	0.027*	70.9 ± 6.5	0.656
OralB	36.0 ± 6.5	9.8 ± 2.4	0.004**	75.4 ± 6.3	0.139
SD	35.4 ± 5.5	9.6 ± 2.2	0.001**	74.4 ± 4.2	0.352
Total (*n* = 36)	35.2 ± 3.2	13.3 ± 2.1	< 0.0001**		
Poor brushing group (*n* = 5)					
Brush	46.3 ± 10.7	29.6 ± 8.9	0.266	35.3 ± 8.5	
AF	32.6 ± 10.4	12.2 ± 5.9	0.137	69.4 ± 9.4	0.144
OralB	33.4 ± 9.3	8.3 ± 6.3	0.053	80.7 ± 6.3	0.008*
SD	30.6 ± 7.2	8.2 ± 2.7	0.032*	75.7 ± 5.5	0.049*
Good brushing group (*n* = 4)					
Brush	29.6 ± 8.2	14.9 ± 5.3	0.193	75.9 ± 2.8	
AF	28.3 ± 10.8	8.9 ± 4.4	0.170	72.7 ± 10.0	> 0.999
OralB	39.2 ± 10.2	11.7 ± 4.1	0.068	68.8 ± 12.1	> 0.999
SD	41.4 ± 8.7	11.5 ± 3.8	0.034*	72.7 ± 7.2	> 0.999
Buccal (*n* = 9)					
Brush	37.3 ± 7.1	22.1 ± 5.1	0.104	52.8 ± 7.9	
AF	27.9 ± 6.3	9.4 ± 3.2	0.023*	70.1 ± 7.3	0.210
OralB	31.1 ± 6.0	7.3 ± 2.4	0.004**	77.7 ± 6.5	0.023*
SD	32.9 ± 6.3	6.9 ± 1.7	0.003**	79.5 ± 3.7	0.042*
Total (*n* = 36)	32.3 ± 3.1	11.4 ± 1.9	< 0.0001**		
Palatal (*n* = 9)					
Brush	42.1 ± 7.7	26.0 ± 7.6	0.143	53.1 ± 10.4	
AF	38.2 ± 9.4	13.8 ± 5.0	0.035*	68.2 ± 7.4	0.879
OralB	44.4 ± 7.8	15.9 ± 4.1	0.007*	71.7 ± 6.0	0.517
SD	43.8 ± 7.8	17.4 ± 4.5	0.002**	66.1 ± 5.7	> 0.999
Total (*n* = 36)	42.3 ± 4.0	18.2 ± 2.7	< 0.0001**		

Unpaired *t* tests with Welch’s correction were performed to compare the two groups. Kruskal-Wallis one-way analysis of variance, followed by Dunn’s multiple comparisons test, was used to determine statistically significant differences. Significant differences are shown as italic numbers. *p < 0.05; **p < 0.005

**Fig. 4:**
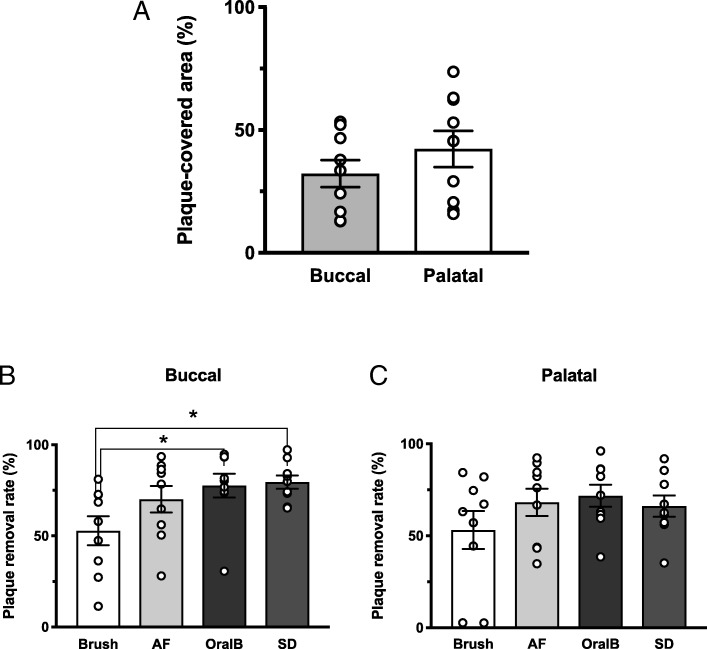
Comparison of plaque in the buccal and palatal areas and plaque removal rates. **a** The plaque-covered areas in the palatal side are larger than those in the buccal side. **b** The SD and OralB plaque removal rates in the buccal area were significantly higher than those of manual brushing; no significant differences were observed among the other instruments. **c** There were no significant differences in the plaque removal rates in the palatal side among instruments. *p <  0.05

Before brushing, plaque covered 32.3% ± 3.1% of the fitting surface in the buccal area and 42.3% ± 4.0% of the fitting surface in the palatal area (Fig. 4a). The SD and OralB groups exhibited significant improvement in plaque removal in the buccal area of the prosthesis, compared with that of the control group. The plaque removal rates for the buccal area of the prosthesis were 52.8 ± 7.9%, 70.1 ± 7.3%, 77.7 ± 6.5%, and 79.5 ± 3.7% for the control, AF, OralB, and SD groups, respectively (Fig. 4b). There were no significant differences in the plaque removal rates in the palatal area among instruments, with rates of 53.1 ± 10.4%, 68.2 ± 7.4%, 71.7 ± 6.0%, and 66.1 ± 5.7% for the control, AF, OralB, and SD groups, respectively (Fig. 4c). The plaque removal rates in the palatal area were consistently lower than those in the buccal area for each of the three electric instruments. Statistical post hoc power was calculated as 0.62 in this study.

## Discussion

There is controversy regarding whether electric-powered toothbrushing is better than manual toothbrushing for maintaining oral hygiene in patients with FDPs [19]. The efficacy of an electric-powered toothbrush has been reported previously; despite limitations regarding variability in study design, use of an electric toothbrush appears to be more effective for plaque removal than manual toothbrushing for both fixed and removable implant-supported prostheses [20–22]. However, little is known about the best type of powered brush for FDPs in edentulous patients, and there is no evidence supporting use of a particular cleaning method for the fitting surface of All-on 4™ concept FDPs. Since the prosthesis is retained by four implants, the area contacting the alveolar ridge between each implant spontaneously becomes a long-fitting surface and thus is likely to become covered with plaque. Abi Nader et al. reported the area of plaque accumulation on the fitting surface of maxillary All-on 4™ FDPs to be 28.3 ± 8.4% [23], which is comparable with our result of 35.2 ± 3.2%. These results suggest that approximately one third of the fitting surfaces of All-on 4™ FDPs are likely to be covered with plaque.

Although our results did not show significant differences among instruments for the entire group of participants, we found that the plaque removal rate significantly improved when the poor brushing group (manual toothbrush) used an electric-powered toothbrush. The good brushing group exhibited little, if any, differences; patients who achieved good plaque removal with the manual brush achieved consistently good outcomes with the electric-powered instruments. These findings suggest that the removal of plaque accumulation can be improved, particularly among patients who are not adept at manual toothbrushing.

When we evaluated the buccal and palatal areas of the fitting surfaces of FDPs, we found that the palatal area was 1.3 times more likely to be covered with plaque than the buccal area. Abi Nader et al. previously reported a similar trend [23]. Although we found that the SD and OralB electric toothbrushes were preferable to the manual toothbrush in the buccal area, there were no significant differences in the palatal area; the plaque removal rates in the buccal area were consistently lower than those in the palatal area for the three electric instruments. These results clearly show that plaque is more likely to accumulate in the palatal area than in the buccal area and that it is more difficult to remove plaque from the palatal area. This is likely because of the anatomical structure of the FDPs and the difficulty in accessing the area with cleaning instruments. Of note, two subjects had extremely low plaque removal rates of 2.6% and 2.7% in the palatal area. These results indicate that it is very difficult for some subjects to achieve adequate oral hygiene in the palatal area of FDPs with a manual toothbrush.

Given that accumulated plaque surrounding an implant is associated with peri-implantitis in partially edentulous [8] and edentulous patients [12], prevention of plaque accumulation is necessary for maintaining healthy mucosa around an implant. Our investigation demonstrated that the plaque removal rate at the fitting surface of an FDP is better with an electric toothbrush than with a manual toothbrush, particularly among those who are not adept at manual toothbrushing. Our results support a recommendation for edentulous patients with FDPs to use electric toothbrushes instead of manual toothbrushes to facilitate plaque removal. Of note, mechanical vibration forces could loosen the occlusal screws of the FDPs; therefore, patients who use electric toothbrushes should be assessed for this issue during follow-up.

The fact that no instruments were able to effectively remove accumulated plaque in the palatal areas of the FDPs suggests that the palatal area of an FDP should be minimized during fabrication and that professional care may be required to maintain hygiene of the prosthesis.

The limitations of this study include its relatively small sample size and the cross-sectional evaluation over a short period of time. Within the limitations of this study, our results demonstrate the favorable effects of using electric toothbrushes, compared with manual toothbrushes, by patients with FDPs in order to achieve long-term stability of the implants.

## Conclusions

We found that the use of electric toothbrushes resulted in better plaque removal rates from the area of the prostheses touching the alveolar ridge than manual toothbrushes, particularly among those who were not adept at manual toothbrushing. These results suggest that electric toothbrush use may be an effective part of a self-performed cleaning protocol for patients with All-on-4™ concept FDPs to facilitate plaque removal.

## Abbreviations

AF: Air Floss® (Koninklijke Philips N.V., Amsterdam, the Netherlands)
Brush: Tuft24® MS (OralCare Inc., Tokyo, Japan)
FDP: Implant-supported fixed dental prosthesis
OralB: Oral-B Professional Care Smart Series 5000® (Braun GmbH, Kronberg, Germany)
SD: Sonicare Diamond Clean® (Koninklijke Philips N.V.)
